# Supplementary material for: Necessary conditions for sustainable water and sanitation service delivery in schools: A systematic review
Source: PLoS One. 2022 Jul 20;17(7):e0270847. doi: 10.1371/journal.pone.0270847 (PMC9299385; doi:10.1371/journal.pone.0270847)
Supplement: S1 Table — (PDF) [file pone.0270847.s001.pdf]

**S1 Table**

**S1 Table. Quality assessments of observational studies.**

| Study | Appropriate qualitative approach? | Clear study aims? | Defensible research design? | Appropriate data collection strategy? | Described role of researcher? | Clearly described context? | Reliable methods? | Rigorous data analysis? | Reliable data analysis? | Findings relevant to aims? | Adequate discussion of limitations? | Clear reporting of ethics? |
|---|---|---|---|---|---|---|---|---|---|---|---|---|
| Chatterley et al. (2013) | + | + | + | + | - | + | + | + | - | + | + | + |
| Chatterley et al. (2014) | + | + | + | + | - | + | + | + | + | + | + | + |
| Okello et al. (2019) | + | + | + | + | + | + | + | + | + | + | + | + |
| Graves et al. (2014) | + | + | + | + | - | - | + | + | + | + | + | + |
| Ikoya, Peter et al. (2008) | + | + | + | + | - | + | + | - | - | + | - | - |
| Mumtaz et al. (2019) | + | + | + | + | - | + | + | + | + | + | + | + |
| Saboori et al. (2011) | + | + | + | + | - | - | + | - | - | + | - | - |
| Xaba, M I (2012) | + | + | + | + | - | + | + | + | - | + | - | + |
| Snyder et al. (2020) | + | + | + | + | - | + | + | + | - | + | + | + |
