# Supplementary material for: Necessary conditions for sustainable water and sanitation service delivery in schools: A systematic review
Source: PLoS One. 2022 Jul 20;17(7):e0270847. doi: 10.1371/journal.pone.0270847 (PMC9299385; doi:10.1371/journal.pone.0270847)
Supplement: S2 Table — (PDF) [file pone.0270847.s002.pdf]

S2 Table

S2 Table. Quality assessment rubric for observational studies.

Appropriate qualitative approach?		Clear study aims?		Defensible research design?		Appropriate data collection?		Described role of researcher?		Clearly described context?	
+	Study asks a "how" or "why" research question.	+	Objectives and/or research questions are explicitly stated.	+	Rationale provided for research design components including sampling, selection of cases, data collection and data analysis techniques.	+	Data collection methods are clearly described and are appropriate.	+	Role and status of researchers are explicitly described.	+	Characteristics of the participants and settings are well-described.
-	Study asks a "how much" or "how many" research question.	-	Objectives and/or research questions are not explicitly stated.	-	Rationale not provided for research design components including sampling, selection of cases, data collection and data analysis techniques.	-	Data collection methods are not clearly described and/or are inappropriate.	-	Role and status of researchers are not explicitly described.	-	Characteristics of the participants and settings are not well-described.
**Reliable methods?**		**Rigorous data analysis?**		**Reliable analysis?**		**Findings relevant to aims?**		**Adequate discussion of limitations?**		**Appropriate reporting of ethics?**	
+	Triangulation of different sources (or justification if lack thereof).	+	Data analysis procedure is explicit, justified, and systematic (e.g. multiple coders, a priori determined code).	+	More than 1 researcher coded the data (transcripts). Information on how differences were resolved was provided.	+	Findings are relevant to objectives and research questions.	+	Explicit discussion of limitations and their impacts on the study results.	+	Explicit discussion of ethics, consent, anonymity, changing behavior, raising expectations, and/or approval from ethics committee.
-	Lack of triangulation and lack of justification for failure to obtain multiple sources.	-	Data analysis procedure is not well-described and justified.	-	Only 1 researcher coded the data. The description of the analysis strategy was inadequate.	-	Findings are not relevant to objectives and research questions.	-	No discussion of limitations.	-	No discussion of any of the above ethical considerations.
