# Supplementary material for: Necessary conditions for sustainable water and sanitation service delivery in schools: A systematic review
Source: PLoS One. 2022 Jul 20;17(7):e0270847. doi: 10.1371/journal.pone.0270847 (PMC9299385; doi:10.1371/journal.pone.0270847)
Supplement: S3 Table — (PDF) [file pone.0270847.s003.pdf]

S3 Table

S3 Table. Quality assessment of quasi-experimental studies.

Study	Well-described source population?	Representa-tive eligible population?	Representa-tive participants?	Selection bias minimized during allocation?	Acceptably low contamination?	Adjusted for confounders?	Reliable outcome measures?	Similar follow-up times in all arms?	Meaningful follow-up time?	Sufficiently powered?	Considered multiple explanatory variables?	Reported precision of effect sizes?
Alexander et al. (2014)	++	-	+	NA	NR	-	+	NA	NA	-	+	++
Karon et al. (2017)	++	-	++	NA	NR	+	+	NA	NA	NR	+	+
Kochurani et al. (2009)	++	-	+	NA	NR	-	-	NA	NA	NR	-	++

[1] NA = not applicable.

[2] NR = not reported.
