# Supplementary material for: Necessary conditions for sustainable water and sanitation service delivery in schools: A systematic review
Source: PLoS One. 2022 Jul 20;17(7):e0270847. doi: 10.1371/journal.pone.0270847 (PMC9299385; doi:10.1371/journal.pone.0270847)
Supplement: S4 Table — (PDF) [file pone.0270847.s004.pdf]

S4 Table

S4 Table. Quality assessment rubric for quasi-experimental studies.

Well-described source population?		Representative eligible population?		Representative participants?		Selection bias minimized during allocation?		Acceptably low contamination?		Adjusted for confounders?	
++	Provided information on school type (public/private), location (urban/rural), country, province, level of schooling (primary, secondary).	++	Recruitment of schools was well-described, and the eligible population appears to be representative of source population.	++	Selection of participants is well described. Inclusion and exclusion criteria are explicit and appropriate. Selected participants appear to be representative of the eligible population.	++	Allocation was randomized and details about how the randomization occurred (e.g. across which attributes, strata) were provided.	++	Comparison group did not receive intervention.	++	Potential confounders are discussed and/or dismissed due to explicit justification. Other likely confounders were considered and adjusted for.
+	Missing information on one of the above population characteristics.	+	Recruit is well-described, but eligible population does not appear to be representative of source population.	+	Inclusion and exclusion criteria are explicit, but the selection of participants is not well-described. Author(s) do not provide sufficient information to assess representativeness.	+	Allocation was randomized, but no details of the randomization were provided.	+	NA	+	Some confounders were controlled for, but other likely confounders were not adjusted for.
-	Missing information on two or more of the above population characteristics.	-	Insufficient information provided on recruitment, and therefore, unable to assess whether the eligible population is representative of the source population.	-	Inclusion and exclusion criteria are not explicitly provided and the selection of participants is not well-described. Insufficient information to asses representativeness.	-	Allocation was not randomized.	-	Comparison group received intervention and it was likely to cause bias.	-	No mention of adjusting for any confounders and no discussion/justification supporting this decision.
**Reliable outcome measures?**		**Similar follow-up times in all arms?**		**Meaningful follow-up time?**		**Sufficiently powered?**		**Considered multiple explanatory variables?**		**Reported precision of effect sizes?**	
++	Well-defined outcomes presented with inter- or intra-rater reliability scores (if applicable). Multiple data collection strategies used to gather evidence (e.g. self-reported and enumerator observations).	++	Yes	++	1 year or more.	++	Provided power calculations demonstrate that the study is sufficiently powered for the outcome(s) of interest.	++	Multiple explanatory variables were considered in the analyses. Alternatively, justification for not including other explanatory variables was provided.	++	Precision of intervention effects provided for all specified outcomes.
+	Incomplete definition of outcomes and/or no intra- or inter-reliability scores reported (if applicable).	+	NA	+	More than 6 months, but less than 1 year.	+	Power calculations are provided, but demonstrate that the study was insufficiently powered.	+	Multiple explanatory variables were considered in some analyses, but potentially important explanatory variables were left out without justification.	+	Precision of intervention effects provided for some specified outcomes .

-	Outcomes were not defined.	-	No	-	Less than 6 months.	-	No power calculations provided.	-	Multiple explanatory variables were not considered in the analyses.	-	Precision of intervention effects not provided.

4
