# Supplementary material for: Necessary conditions for sustainable water and sanitation service delivery in schools: A systematic review
Source: PLoS One. 2022 Jul 20;17(7):e0270847. doi: 10.1371/journal.pone.0270847 (PMC9299385; doi:10.1371/journal.pone.0270847)
Supplement: S5 Table — (PDF) [file pone.0270847.s005.pdf]

S5 Table

S5 Table. Quality assessment of experimental studies.

Ref	Well-described source population?	Representative eligible population?	Representative participants?	Selection bias minimized during allocation?	Well-described interventions?	Allocation concealed?	Blinding?	Adequate exposure to treatment?	Low contamination	Similar treatment of groups?
Alexander et al. (2013)	-	-	+	+	+	NR	NR	+	NR	++
Alexander et al. (2018)	+	-	+	+	+	NR	NR	NR	NR	++
Bohnert et al. (2016)	++	-	+	++	+	++	-	-	NR	-
Booysen, MJ (2019)	-	-	-	-	-	NA	NA	NR	NA	NA
Buxton et al. (2019)	+	-	+	++	+	++	-	-	NR	++
Caruso et al. (2014)	+	-	+	++	+	++	-	NR	NR	++
Saboori et al. (2013)	++	-	+	++	+	NR	-	NR	NR	++

Ref	Adjusted for confounders?	High participant retention?	Reliable outcome measures?	Similar follow-up in treatment and control arms?	Meaningful follow-up?	Similar baseline across arms?	Sufficiently powered?	Reported effect sizes?	Reported precision of effect sizes?	
Alexander et al. (2013)	-	++	++	++	-	-	NR	++	++	
Alexander et al. (2018)	-	++	+	++	++	++	NR	++	++	
Bohnert et al. (2016)	-	+	+	-	+	-	NR	++	++	
Booysen, MJ (2019)	NA	++	+	++	-	NA	NR	++	-	
Buxton et al. (2019)	++	+	+	++	-	+	NR	++	++	
Caruso et al. (2014)	++	++	++	++	-	++	+	++	++	

Saboori et al. (2013)	++	++	++	++	-	++	+	++	++	

[1] NR = not reported.
[2] NA = not applicable.
