# Supplementary material for: Necessary conditions for sustainable water and sanitation service delivery in schools: A systematic review
Source: PLoS One. 2022 Jul 20;17(7):e0270847. doi: 10.1371/journal.pone.0270847 (PMC9299385; doi:10.1371/journal.pone.0270847)
Supplement: S6 Table — (PDF) [file pone.0270847.s006.pdf]

S6 Table

S6 Table. Quality assessment rubric for experimental studies.

Well-described source population?		Representative eligible population?		Representative participants?		Selection bias minimized during allocation?		Well-described interventions?		Allocation concealed?	
++	Provided information on school type (public/private), location (urban/rural), country, province, level of schooling (primary, secondary).	++	Recruitment of schools was well-described, and the eligible population appears to be representative of source population.	++	Selection of participants is well described. Inclusion and exclusion criteria are explicit and appropriate. Selected participants appear to be representative of the eligible population.	++	Allocation was randomized and details about how the randomization occurred (e.g. across which attributes, strata) were provided.	++	Sufficient details about the intervention were provided such that the intervention could be replicated.	++	Allocation was randomized using a computer.
+	Missing information on one of the above population characteristics.	+	Recruit is well-described, but eligible population does not appear to be representative of source population.	+	Inclusion and exclusion criteria are explicit, but the selection of participants is not well-described. Author(s) do not provide sufficient information to assess representativeness.	+	Allocation was randomized, but no details of the randomization were provided.	+	At least one key interventional detail was missing.	+	NA
-	Missing information on two or more of the above population characteristics.	-	Insufficient information provided on recruitment, and therefore, unable to assess whether the eligible population is representative of the source population.	-	Inclusion and exclusion criteria are not explicitly provided and the selection of participants is not well-described. Insufficient information to asses representativeness.	-	Allocation was not randomized.	-	Many key intervention details were missing.	-	Allocation was not randomized by a computer
**Blinding?**		**Adequate exposure to treatment?**		**Low contamination**		**Similar treatment of groups?**		**Adjusted for confounders?**		**High participant retention?**	
++	Participants and investigators were double or triple blinded.	++	High intervention fidelity and exposure.	++	Comparison group did not receive intervention.	++	Groups were treated equally.	++	Potential confounders are discussed and/or dismissed due to explicit justification. Other likely confounders were considered and adjusted for.	++	All participants accounted for.
+	Lack of blinding is unlikely to cause bias.	+	Variable intervention fidelity and exposure is unlikely to bias the results.	+		+	Groups were treated differently, but this is unlikely to cause bias.	+	Some confounders were controlled for, but other likely confounders were not adjusted for.	+	Some participants were lost, but this is unlikely to cause bias.

-	Lack of blinding is likely to cause bias.	-	Low intervention fidelity and exposure is likely to bias the results.	-	Comparison group received intervention and this is likely to cause bias.	-	Groups were treated differently, and this is likely to cause bias.	-	No mention of adjusting for any confounders and no discussion/justification supporting this decision.	-	Participant s were lost and this is likely to cause bias.
Reliable outcome measures?		Similar follow-up in treatment and control arms?		Meaningful follow-up?		Similar baseline across arms?		Sufficiently powered?		Reported effect sizes?	
++	Well-defined outcomes presented with inter- or intra-rater reliability scores (if applicable). Multiple data collection strategies used to gather evidence (e.g. self-reported and enumerator observations).	++	Yes	++	1 year or more.	++	Balanced baseline and/or adjusted for unbalanced baseline. P-values were provided to assess balance.	++	Provided power calculations demonstrate that the study is sufficiently powered for the outcome(s) of interest.	++	Effect sizes provided.
+	Incomplete definition of outcomes and/or no intra- or inter-reliability scores reported (if applicable).	+		+	More than 6 months, but less than 1 year.	+	Mostly balanced baseline and no adjustments, but the differences are unlikely to cause bias. P-values were provided or statistical tests to assess balance were discussed.	+	Power calculations are provided, but demonstrate that the study was insufficiently powered.	+	
-	Outcomes were not defined.	-	No	-	Less than 6 months.	-	Unbalanced baseline is likely to cause bias. Alternatively, p-values and statistical tests were not provided nor discussed to assess balance.	-	No power calculations provided.	-	Effect sizes not provided.
Reported precision of effect sizes?											
++	Precision of intervention effects provided for all specified outcomes.										
+	Precision of intervention effects provided for some specified outcomes .										
-	Precision of intervention effects not provided.										

[1] NA = not applicable.
