# Supplementary material for: Necessary conditions for sustainable water and sanitation service delivery in schools: A systematic review
Source: PLoS One. 2022 Jul 20;17(7):e0270847. doi: 10.1371/journal.pone.0270847 (PMC9299385; doi:10.1371/journal.pone.0270847)
Supplement: S7 Table — (PDF) [file pone.0270847.s007.pdf]

S8 Table

S8 Table. Indicator definitions and data collection details associated with experimental and quasi-experimental studies that evaluated maintenance outcomes pertaining to water facilities.

Observed and reported   Reported   Observed

Water facilities			
**Study**	**Availability of drinking water**	**Functionality of drinking water source**	**Data Collection Details**
Alexander et al. (2013)	A binary indicator coded as "yes" if drinking water was observed to be available at the time of data collection.	A binary indicator coded as "yes" if at least one water storage container had a functioning tap at the time of data collection.[1]	Trained enumerators visited schools at four unannounced follow-up times and performed spot checks to observe infrastructure while administering the survey to a head-teacher.
Alexander et al. (2014)	Not Measured	Not Measured	Trained enumerators visited schools at one unannounced follow-up time and performed structured observation of infrastructure.
Alexander et al. (2018)	Not Measured	Not Measured	-
Bohnert et al. (2016)	Not Measured	Not Measured	-
Booysen, MJ (2019)	Not Measured	A continuous indicator, minimum nightly flow (MNF), was used to identify any abnormal water flow that might indicate leaks.	A real-time water usage meter was installed on the main municipal meter of each school. A pulse sensor recorded the number of pulses in a day, representing a defined volume of flow, and transmitted data to a cloud server.
Buxton et al. (2019)	Not Measured	Not Measured	-
Caruso et al. (2014)	Not Measured	Not Measured	-

Saboori et al. (2013)	Not Measured	Not Measured	-
Karon et al. (2017)	Not Measured	A binary indicator coded as "yes" if water was observed to be available at the source at the time of data collection.<br><br>A binary indicator for functionality in the dry season coded as "yes" if a school administrator reported "water available" at the source for 5-7 days per week during the dry season.	Trained enumerators and supervisors visited schools at one time point wherein enumerators conducted interviews with students and supervisors collected data on school hardware through interviews with a school administrator and observation.
Kochurani et al. (2009)	A binary indicator coded as "yes" if a school reported having water every day.	Not Measured	Teams of two enumerators made unannounced visits to schools to where they observed sanitation facilities for their maintenance and cleanliness. Interviews with teachers, students, and small groups of students were also performed to collect data about use of facilities and hygiene behaviors.

[1] The authors did not differentiate between water storage containers for drinking water *versus* handwashing water. Therefore, we reported the same indicator for "Functionality of drinking water facilities" and "Functionality of handwashing facilities."
