# Supplementary material for: Necessary conditions for sustainable water and sanitation service delivery in schools: A systematic review
Source: PLoS One. 2022 Jul 20;17(7):e0270847. doi: 10.1371/journal.pone.0270847 (PMC9299385; doi:10.1371/journal.pone.0270847)
Supplement: S8 Table — (PDF) [file pone.0270847.s008.pdf]

S9 Table

S9 Table. Indicator definitions and data collection details associated with experimental and quasi-experimental studies that evaluated maintenance outcomes pertaining to handwashing facilities.

Observed and reported   Reported   Observed

Handwashing (HW) facilities				
**Study**	**Functionality of handwashing facilities**	**Availability of handwashing water**	**Availability of soap**	**Data Collection Details**
Alexander et al. (2013)	A binary indicator coded as "yes" if at least one water storage container had a functioning tap at the time of data collection. [1]	A binary indicator coded as "yes" if handwashing water was available to students at handwashing stations at the time of data collection.	A binary indicator coded as "yes" if soap was available to students at handwashing stations at the time of data collection.	Trained enumerators visited schools at four unannounced follow-up times and performed spot checks to observe infrastructure while administering the survey to a head-teacher.
Alexander et al. (2014)	Not measured	A binary indicator coded as "yes" if handwashing water was available to students at the time of data collection.<br><br>A binary indicator coded as "yes" if the head teacher reported handwashing water was available the day enumerators visited.<br><br>A binary indicator coded as "yes" if the head teacher reported handwashing water was always available.	A binary indicator coded as "yes" if soap was available to students at the time of data collection.<br><br>A categorical indicator coded as "always", "sometimes", or "never" based on the head teacher's response to if "soap is provided at school."	Trained enumerators visited schools at one unannounced follow-up time and performed structured observation of infrastructure in addition to head-teachers reporting availability of WASH materials. Head teachers also provided information to the best of their knowledge on the availability of soap and water for handwashing.
Alexander et al. (2018)	Not measured	A binary indicator coded as "yes" if handwashing water was observed to be	A binary indicator coded as "yes" if soap was observed to be available to students	Trained enumerators visited schools at five unannounced follow-up times and independently observed WASH conditions in

		available to students at the time of data collection.<br><br>A binary indicator coded as "yes" if the head teacher reported "handwashing water available today."<br><br>A binary indicator coded as "yes" if the head teacher reported "the school always supplies handwashing water."	at the time of data collection.<br><br>A binary indicator coded as "yes" if the head teacher reported "soap available today."<br><br>A binary indicator coded as "yes" if the head teacher reported "the school always supplies soap."	addition to semi-structures interviews with head-teachers or designated representatives on school WASH conditions.
Bohnert et al. (2016)	Not measured	A binary indicator coded as "yes" if handwashing water was present at the beginning of the day of data collection.	A binary indicator coded as "yes" if soap was present at the beginning of the day of data collection.	Trained enumerators visited schools at six unannounced follow-up times and performed structured observation of school WASH facilities in addition to head teacher interviews and environmental swabs or hand rinses.
Booysen, MJ (2019)	Not measured	Not measured	Not measured	-
Buxton et al. (2019)	Not measured	Not measured	Not measured	-
Caruso et al. (2014)	Not measured	A binary indicator coded as "yes" if handwashing water was present at the beginning of a 30 minute recess period during the school day.	A binary indicator coded as "yes" if soap/soapy water was present at the beginning of a 30 minute recess period during the school day.	Trained enumerators visited schools at five follow-up times to observe latrine and handwashing conditions.
Saboori et al. (2013)	Not measured	A binary indicator coded as "yes" if handwashing water was observed to be present at the time of data	A binary indicator coded as "yes" if soap was observed to be present at the time of data collection.	Trained enumerators visited schools at seven follow-up times to perform structured interviews with students and structured observations of school WASH facilities. 30 pupils in grades 4

		collection.<br><br>A binary indicator coded as "yes" if students reported "water always enough for handwashing" during student interviews.	A binary indicator coded as "yes" if students reported "soap always available to wash hands" during student interviews.	through 7 were administered structured interviews at each school to assess pupil perceptions of school handwashing conditions.
Karon et al. (2017)	Not measured	A binary indicator coded as "yes" if water was observed at one or more handwashing stations at the time of data collection.	A binary indicator coded as "yes" if soap was observed at one or more handwashing stations at the time of data collection.	Trained enumerators and supervisors visited schools at one time point wherein enumerators conducted interviews with students and supervisors collected data on school hardware through interviews with a school administrator and observation.
Kochurani et al. (2009)	Not measured	Not measured	A binary indicator coded as "yes" if soap was observed inside the handwashing facility at the time of data collection.	Teams of two enumerators made unannounced visits to schools to where they observed sanitation facilities for their maintenance and cleanliness.

[1] The authors did not differentiate between water storage containers for drinking water *versus* handwashing water. Therefore, we reported the same indicator for "Functionality of drinking water facilities" and "Functionality of handwashing facilities."”
