# Supplementary material for: Necessary conditions for sustainable water and sanitation service delivery in schools: A systematic review
Source: PLoS One. 2022 Jul 20;17(7):e0270847. doi: 10.1371/journal.pone.0270847 (PMC9299385; doi:10.1371/journal.pone.0270847)
Supplement: S9 Table — (PDF) [file pone.0270847.s009.pdf]

S10 Table

S10 Table. Indicator definitions and data collection details associated with experimental and quasi-experimental studies that evaluated maintenance outcomes pertaining to sanitation facilities.

Observed and reported — Reported — Observed

Sanitation facilities						
**Study**	**Accessibility of latrines**	**Structural integrity and functionality of latrines**	**Latrine privacy**	**Latrine cleanliness**	**Supplies for latrine cleaning**	**Data Collection Details**
Alexander et al. (2013)	Not measured	A binary indicator coded as "yes" if a latrine stall door closed completely and had a lock on the inside.	A binary indicator coded as "yes" if a latrine stall had a door.	A continuous indicator defined by observing cleanliness characteristics (smell, feces, internal cleanliness, pooled water, and/or urine) on a scale from 0 (absent) to 2 (very smelly, lots of feces, etc.) and summing to create a cleanliness score ranging from 0 to 8.	Not measured	Trained enumerators visited schools at four unannounced follow-up times and performed spot checks to observe infrastructure while administering the survey to a head-teacher.
Alexander et al. (2014)	Not measured	"Good" latrine structural integrity used as a binary indicator and coded as "yes" if latrine had a roof, walls with no holes, a door and stable floor slab.	A binary indicator coded as "yes" if door had a lock AND had "good" structural integrity. (see definition on left)	A binary indicator coded "yes" if there was lack of strong smell, clean floor AND had "good" structural integrity and was private (see definition on left)	A binary indicator coded as "yes" if any latrine cleaning supplies (such as disinfectant, detergent, brooms) were present at the school.	Trained enumerators visited schools at one unannounced follow-up time and performed structured observation of infrastructure.

Alexander et al. (2018)	Not measured	"Acceptable" latrine was used as a binary indicator and coded as "yes" if latrine was: clean (no visible feces on floor), had no strong/offensive smell, contained door and roof, had no major holes in walls and had stable floor or stable latrine slab.	A binary indicator coded as "yes" at the school level if there was a washing or changing room designated for girls, which at minimum comprised of one girls' stall (toilet or changing room) with a lockable door.	A binary indicator coded as "yes" if a latrine had no visible feces or urine on the floor.	A binary indicator coded as "yes" if any latrine cleaning supplies were present at the school at the time of data collection.<br><br>A binary indicator coded as "yes" if the head teacher reported "latrine cleaning supplies available today" on the day enumerators visited.<br><br>A binary indicator coded as "yes" if the head teacher reported "school always had latrine cleaning supplies."	Trained enumerators visited schools at five unannounced follow-up times and independently observed WASH conditions in addition to semi-structures interviews with head-teachers or designated representatives on school WASH conditions.
Bohnert et al. (2016)	A binary indicator coded as "yes" if a latrine door was unlocked at the time of data collection.	Not measured	Not measured	A continuous indicator defined by structured observation of five binary (yes/no) cleanliness indicators: availability of cleaning materials, absence of flies, no odor, no visible feces, and absence of urine/stagnant water. Affirmative responses were assigned one point, and values were summed to create a scale of toilet maintenance ranging from 0 (very dirty) to 5	One of five binary indicators evaluated as part of the toilet cleanliness/maintenance score coded as "yes" if cleaning materials were observed to be available.	Trained enumerators visited schools at six unannounced follow-up times and performed structured observation of school WASH facilities in addition to head teacher interviews and environmental swabs or hand rinses.

				(very clean).<br><br>A continuous indicator defined by head teachers reporting the weekly frequency of toilet cleaning.		
Booysen, MJ (2019)	Not measured	Not measured	Not measured	Not measured	Not measured	-
Buxton et al. (2019)	A binary indicator coded as "yes" if a latrine door was unlocked to students.	A binary indicator coded as "yes" if water was available to students for flushing.	A binary indicator coded as "yes" if all of the following criteria were observed: (1) presence of a door/curtain, (2) that closes completely, (3) can be locked from the inside, and (4) has no large gaps/holes in the structure.	A binary indicator defined by structured observation of 8 binary (yes/no) quality indicators: odour; faeces on walls/ floor; flies; lighting; urine or stagnant water on floor; mud on the floor; faeces in the bowl; and visible litter. Scores were summed and scaled to yield a score out of 10. Toilets which scored at or above 8.5/10 were coded as "high quality."	Not measured	Enumerators visited schools at baseline and endline to observe school WASH conditions specified in the Toilet Usability Index (TUX) developed prior to this intervention to evaluate toilet usability.
Caruso et al. (2014)	Not measured	Not measured	A binary indicator coded as "yes" if a latrine stall had a door.	A continuous indicator defined by assessing 5 latrine conditions and rating from 0 (absence) to 2 (strong presence): presence of faeces, urine, flies, smell, and mud. Scores were summed	Not measured	Trained enumerators visited schools at five follow-up times to observe latrine and handwashing conditions.

				to yield a score out of 10.		
Saboori et al. (2013)	Not measured	Not measured	Not measured	Not measured	Not measured	-
Karon et al. (2017)	A binary indicator coded as "yes" if one or more toilets were observed to be accessible for disabled students at the time of data collection.	A binary indicator coded as "yes" if one or more toilets per 100 students were observed to be functional.<br><br>A binary indicator coded as "yes" if one or more toilets were observed to be non-functional at the time of data collection.	Not measured	A categorical variable coded as "not clean", "somewhat clean", or "clean." Clean was defined as the absence of odor, visible feces, flies, and litter. Somewhat clean was defined as some smell and/or some sign of fecal matter and/or some flies and/or some litter. Not clean was defined as the presence of a strong odor and/or fecal matter.	Not measured	Trained enumerators and supervisors visited schools at one time point wherein enumerators conducted interviews with students and supervisors collected data on school hardware through interviews with a school administrator and observation.
Kochurani et al. (2009)	Not measured	A continuous indicator defined by observation of three criteria: all cubicles have door and latch, all bowls have water seal in place, all toilets have functional pit (not full or leaking). Schools were given 1 point for each criteria fulfilled and points were	Not measured	A binary indicator coded as "yes" at the school level if all pan/toilet slabs were observed to be free from visible excreta and all urinals were free from visible urine pools at the time of data collection.	Not measured	Teams of two enumerators made unannounced visits to schools to where they observed sanitation facilities for their maintenance and cleanliness.

		summed with a maximum score of 3.				
