# Supplementary material for: Necessary conditions for sustainable water and sanitation service delivery in schools: A systematic review
Source: PLoS One. 2022 Jul 20;17(7):e0270847. doi: 10.1371/journal.pone.0270847 (PMC9299385; doi:10.1371/journal.pone.0270847)
Supplement: S10 Table — (PDF) [file pone.0270847.s010.pdf]

S11 Table

S11 Table. Reported outcome statistics from experimental and quasi-experimental studies that implemented interventions with infrastructure maintenance components.

(green)	Significant improvement ($p \leq 0.05$)
(yellow)	Non-significant impact ($p > 0.05$)
(red)	Significant deterioration ($p \leq 0.05$)
(black)	No statistical significance reported
(light grey)	Not measured

Study	Intervention Arm	Indicator Type	Functionality of drinking water facilities	Functionality of handwashing facilities	Accessibility of latrines	Structural integrity and functionality of latrines	Latrine privacy	Latrine cleanliness
Alexander et al. (2013)	Budget	Observed	p=0.015 of Poisson regression coefficient of difference between intervention and control. [1]	p=0.015 of Poisson regression coefficient of difference between intervention and control. [1]		p=0.013 of Poisson regression coefficient of difference between intervention and control.	p=0.643 of Poisson regression coefficient of difference between intervention and control.	p<0.001 of Poisson regression coefficient of difference between intervention and control.
	Accountability	Observed	p<0.001 of Poisson regression coefficient of difference between intervention and control. [1]	p<0.001 of Poisson regression coefficient of difference between intervention and control. [1]		p=0.706 of Poisson regression coefficient of difference between intervention and control.	p=0.097 of Poisson regression coefficient of difference between intervention and control.	p<0.001 of Poisson regression coefficient of difference between intervention and control.
	Maintenance	Observed	p=0.015 of Poisson regression coefficient of difference between	p=0.015 of Poisson regression coefficient of difference between		p<0.001 of Poisson regression coefficient of difference between	p=0.433 of Poisson regression coefficient of difference between	p<0.001 of Poisson regression coefficient of difference between

			intervention and control. [1]	intervention and control. [1]		intervention and control.	intervention and control.	intervention and control.
Alexander et al. (2014)	Latrine construction	Observed				p=0.21 for Yates uncorrected chi-squared test between intervention and non-intervention schools.	p=0.37 for Yates uncorrected chi-squared test between intervention and non-intervention schools.	p=1.00 for Yates uncorrected chi-squared test between intervention and non-intervention schools.
	Water and handwashing	Observed						p=0.05 for Yates uncorrected chi-squared test between intervention and non-intervention schools. [2]
Alexander et al. (2018)	Treatment	Observed				p=0.0046 for Paired t-test for change in conditions at all intervention schools from baseline to follow up.	p=0.0100 for Paired t-test for change in conditions at all intervention schools from baseline to follow up.	p=0.0029 for Paired t-test for change in conditions at all intervention schools from baseline to follow up.

Bohnert et al. (2016)	Treatment	Observed			p=0.46 for difference between intervention arms.			p=0.002 for difference between intervention arms.
		Reported						p=0.99 for difference between intervention arms.
Booysen, MJ (2019)	Treatment	Observed	28% average reduction in minimum nightly flow among intervention schools. No p-value reported.					
Buxton et al. (2019)	Treatment	Observed			p=0.737 for difference from baseline to endline between intervention and controls schools.	p=0.271 for difference from baseline to endline between intervention and controls schools.	p=0.280 for difference from baseline to endline between intervention and controls schools.	p=0.660 for difference from baseline to endline between intervention and controls schools.
Caruso et al. (2014)	Latrine cleaning and Handwashing	Observed					p=0.47 for comparison of intervention versus control arms adjusting for baseline values.	p=0.01 for comparison of intervention versus control arms adjusting for baseline values.

	Handwashing	Observed					p=0.13 for comparison of intervention versus control arms adjusting for baseline values.	p=0.11 for comparison of intervention versus control arms adjusting for baseline values.
Saboori et al. (2013)	Latrine Cleaning and Handwashing	NA						
	Handwashing	NA						
Karon et al. (2017)	Treatment	Observed	No statistically significant difference was detected between intervention and non-intervention schools. No p-value reported.		No comparative statistic reported.	No statistically significant difference was detected between intervention and non-intervention schools. No p-value reported.		p=0.02 difference in number of schools having at least one clean toilet between intervention and non-

						No statistically significant difference was detected between intervention and non-intervention schools. No p-value reported.		intervention schools.
		Reported	No comparative statistic reported.					
Kochurani et al. (2009)	Treatment	Observed				Toilets in intervention schools had better toilet maintenance scores than non-intervention schools[3].		Toilets in intervention schools were cleaner than non-intervention schools. No p-value reported.

[1] The authors did not differentiate between water storage containers for drinking water *versus* handwashing water. Therefore, we reported the same statistic for "Functionality of drinking water facilities" and "Functionality of handwashing facilities."

[2] The authors reported a p-value for "Latrine cleanliness" that incorporated structural integrity, privacy, and cleanliness into a single indicator. Individual p-values for "Structural integrity and functionality of latrines" and "Latrine privacy" were not reported.

[3] Study reports $p<0.0001$, however, authors caution that reported p-values are only indicative.
