# Supplementary material for: Necessary conditions for sustainable water and sanitation service delivery in schools: A systematic review
Source: PLoS One. 2022 Jul 20;17(7):e0270847. doi: 10.1371/journal.pone.0270847 (PMC9299385; doi:10.1371/journal.pone.0270847)
Supplement: S11 Table — (PDF) [file pone.0270847.s011.pdf]

S12 Table

S12 Table. Reported outcome statistics from experimental and quasi-experimental studies that implemented interventions with consumables provision components.

Legend	
Significant improvement ($p \leq 0.05$)	Green
Non-significant impact ($p > 0.05$)	Yellow
Significant deterioration ($p \leq 0.05$)	Red
No statistical significance reported	Black
Not measured	Light grey

Study	Intervention Arm	Indicator Type	Availability of drinking water	Availability of handwashing water	Availability of soap	Supplies for latrine cleaning
Alexander et al. (2013)	Budget	Observed	$p=0.015$ of Poisson regression coefficient of difference between intervention and control.	$p=0.003$ of Poisson regression coefficient of difference between intervention and control.	$p<0.001$ of Poisson regression coefficient of difference between intervention and control.	
	Accountability	Observed	$p<0.001$ of Poisson regression coefficient of difference between intervention and control.	$p<0.001$ of Poisson regression coefficient of difference between intervention and control.	$p<0.001$ of Poisson regression coefficient of difference between intervention and control.	
	Maintenance	Observed	$p=0.015$ of Poisson regression coefficient of difference between intervention and control.	$p=0.01$ of Poisson regression coefficient of difference between intervention and control.	$p<0.001$ of Poisson regression coefficient of difference between intervention and control.	
	Latrine construction	NA				

Alexander et al. (2014)	Water and handwashing	Observed		p<0.001 for Yates uncorrected chi-squared test between intervention and non-intervention schools.	No comparative statistic reported.	No comparative statistic reported.
		Reported		p=0.07 for Yates uncorrected chi-squared test between intervention and non-intervention schools.	No comparative statistic reported.	No comparative statistic reported.
				p=0.03 for Yates uncorrected chi-squared test between intervention and non-intervention schools.		
Alexander et al. (2018)	Treatment	Observed		p=0.0548, p=0.0319, p=0.3434 for Paired t-test for change in availability within 3 intervention groups from baseline to average follow up. [1]	p=0.0037, p=0.0165, p=0.0030 for Paired t-test for change in availability within 3 intervention groups from baseline to average follow up. [1]	No comparative statistic reported.
		Reported		p=1.000, p=0.6637, p=0.3938 for Paired t-test for change in availability within 3 intervention groups from baseline to average follow up. [1]	p<0.001, p=0.0296, p<0.001 for Paired t-test for change in availability within 3 intervention groups from baseline to average follow up. [1]	p=0.0550, p=0.0710, p=0.2647 for Paired t-test for change in availability within 3 intervention groups from baseline to average follow up. [1]
				p=0.1679, p=0.3434, p=0.1679 for Paired t-test for change in availability within 3 intervention groups	p=0.0017, p=0.0009, p=0.0011 for Paired t-test for change in availability within 3 intervention	p=0.0852, p=0.0343, p=0.0629 for Paired t-test for change in availability within 3 intervention

				from baseline to average follow up. [1]	groups from baseline to average follow up. [1]	groups from baseline to average follow up. [1]
Bohnert et al. (2016)	Treatment	Observed				No comparative statistic reported.
Booysen, MJ (2019)	Treatment	NA				
Buxton et al. (2019)	Treatment	NA				
Caruso et al. (2014)	Latrine cleaning and handwashing	Observed		p=0.17 for comparison of intervention (average of 4 follow up visits) versus control arms adjusting for baseline values.	p<0.01 for comparison of intervention (average of 4 follow up visits) versus control arms adjusting for baseline values.	
	Handwashing	Observed		p=0.27 for comparison of intervention (average of 4 follow up visits) versus control arms adjusting for baseline values.	p<0.01 for comparison of intervention (average of 4 follow up visits) versus control arms adjusting for baseline values.	
Saboori et al. (2013)	Latrine cleaning and handwashing	Observed		p=0.07 for comparison of intervention (average of 4 follow up visits) versus control arm using logistic regression.	p<0.0001 for comparison of intervention (average of 4 follow up visits) versus control arm using logistic regression.	
		Reported		p=0.32 for comparison of intervention (at final follow up visit) versus control arm using a student t-test.	p<0.0001 for comparison of intervention (at final follow up visit) versus control arm using a student t-test.	

	Handwashing	Observed		p=0.31 for comparison of intervention (average of 4 follow up visits) versus control arm using logistic regression.	p<0.0001 for comparison of intervention (average of 4 follow up visits) versus control arm using logistic regression.	
		Reported		p=0.03 for comparison of intervention (at final follow up visit) versus control arm using a student t-test.	p<0.0001 for comparison of intervention (at final follow up visit) versus control arm using a student t-test.	
Karon et al. (2017)	Treatment	Observed		p=0.002 for comparison of intervention versus non-intervention arm.	p=0.003 for comparison of intervention versus non-intervention arm.	
Kochurani et al. (2009)	Treatment	Observed	Soap was not more available in intervention schools compared to non-intervention schools[2].		Soap was more available in intervention schools than non-intervention schools, but rare in all schools (1-8% of schools)[3].	

[1] P-values from intervention groups are reported together since intervention arms (provision of different types of menstrual hygiene products) are not expected to impact Infrastructure Maintenance or WASH Consumables outcomes.

[2] Study reports p=0.23, however, authors caution that reported p-values are only indicative.

[3] Study reports p=0.01, however, authors caution that reported p-values are only indicative.
