# Supplementary material for: Necessary conditions for sustainable water and sanitation service delivery in schools: A systematic review
Source: PLoS One. 2022 Jul 20;17(7):e0270847. doi: 10.1371/journal.pone.0270847 (PMC9299385; doi:10.1371/journal.pone.0270847)
Supplement: S1 Protocol — (PDF) [file pone.0270847.s012.pdf]

To enable PROSPERO to focus on COVID-19 submissions, this registration record has undergone basic automated checks for eligibility and is published exactly as submitted. PROSPERO has never provided peer review, and usual checking by the PROSPERO team does not endorse content. Therefore, automatically published records should be treated as any other PROSPERO registration. Further detail is provided here.

Citation

Christine Pu, Jenna Davis, Gary Darmstadt. Water, sanitation, and hygiene (WASH) infrastructure maintenance in low- and middle-income countries. PROSPERO 2020 CRD42020199163 Available from: https://www.crd.york.ac.uk/prospero/display_record.php?ID=CRD42020199163

Review question

(1) What are the underlying causes of poorly maintained water, sanitation, and hygiene (WASH) infrastructure at schools in low- and middle-income countries?

(2) What are the enablers and barriers associated with ensuring that students have access to (a) functional and clean WASH facilities and (b) reliable access to WASH consumables (e.g. soap)?

(3) What types of interventions have been tried? What are the conditions under which they are effective in creating an enabling WASH environment?

Searches

An initial electronic search was conducted in English in May 2020 across Scopus, Web of Science, and PubMed to validate the strategy. No restrictions on language and publication period were imposed. Searches will not be re-run prior to the final analysis, but alerts for new studies have been set up and closely monitored for each search engine. Authors of selected papers will be invited to share new and/or unpublished papers relevant to the objectives of this review.

Types of study to be included

Studies of any research design will be included.

Condition or domain being studied

(1) Functionality and cleanliness of WASH infrastructure (e.g. drinking water stations, handwashing stations, latrines, etc.)

(2) Provision of WASH consumables (e.g. soap, water, cleaning supplies, water treatment supplies, etc.)

Participants/population

Inclusion: Public or private educational institutions catering to students in grades K-12 in low- and middle-income countries

Exclusion: Post-secondary education institutions (e.g. colleges and universities); K-12 schools in high-income countries

Intervention(s), exposure(s)

Interventions were of interest if they focused on the maintenance of water, sanitation, and hygiene (WASH) infrastructure in schools. WASH infrastructure was defined to include any built facility that provided water, sanitation, and/or hygiene services. Schools were defined as public or private educational institutions that catered to students in grade 12 and below. Maintenance was defined as any activity that kept the WASH infrastructure functional and clean, either preventatively or in response to repair needs. The provision of consumables such as soap and water were included in the definition of maintenance as they are necessary for functionality. Examples of interventions include: providing schools with cash transfers to address minor infrastructure repairs at schools, providing schools with infrastructure monitoring resources, providing schools with technical maintenance expertise, and providing schools with WASH consumables (cleaning

supplies, soap, etc.).

Comparator(s)/control

Non-exposed control group: schools that did not receive an intervention

Separate intervention arm: schools that received a distinct intervention

Context

Experimental studies were eligible if they implemented an intervention with the explicit objective of increasing the functionality of WASH facilities or increasing the availability and accessibility of WASH consumables. Observational and qualitative studies were eligible if they explored enablers or barriers associated with maintaining the functionality of school WASH infrastructure or with ensuring the availability and accessibility of WASH consumables. Peer-reviewed studies published in English and after 2000 (inclusive) were eligible for inclusion. Eligible studies must have been conducted in a school setting in a low- or middle-income country (LMIC). No other geographic or population restrictions were applied.

Main outcome(s)

(1) Proportion of schools where WASH supplies (most common: disinfectant, soap, chlorine, buckets, and brooms) were (i) observed by an enumerator upon arrival and/or (ii) reported by students and teachers

(2) Proportion of schools where handwashing water and drinking water were (i) observed by an enumerator upon arrival and/or (ii) reported by students and teachers

(3) Latrine cleanliness (various definitions, but often measured by converting enumerator observations of smell, presence of feces and urine, etc. into an aggregate score)

(4) Latrine functionality (various definitions, but often measured by converting enumerator observations into an aggregate score)

(4) Establishing the barriers and facilitators to effective school WASH infrastructure maintenance

Measures of effect

Relative risks, odds ratios, etc. depending on the study and research design

Additional outcome(s)

None

Measures of effect

None

Data extraction (selection and coding)

All search results from the three search engines will be uploaded to Covidence. These search results will be deduplicated. Two researchers will independently screen the titles and abstracts of the search results for eligibility using a standard protocol developed by the authors. For every 650 references screened, two researchers will convene to resolve any conflict decisions. A third researcher will be involved if necessary. The two researchers will discuss until consensus is reached. A similar protocol will be followed for full-text screening. These two screening stages will be completed on Covidence.

Two researchers will use a data extraction template in Excel to collect information from all eligible studies. Extracted information will include details about the study population, study design, implemented intervention (if applicable), data collection strategies, key findings, limitations, and recommendations for future research. The two researchers will convene to discuss and resolve any disagreements. A third researcher will be involved if necessary.

Risk of bias (quality) assessment

Study quality will be assessed using a set of tools developed by the National Institute for Health and Care

Excellence (NICE). Separate quality assessments and tools will be used to evaluate (1) experimental studies, (2) observational studies, and (3) qualitative studies. Experimental and observational studies will be evaluated based on their population selection, method of allocation to intervention, selection and measurement of outcomes, analysis methods and results, and internal and external validity. Qualitative studies will be evaluated based on their research design, data collection methods, communication of the context, analysis methods, and reporting of ethics. Two reviewers will independently evaluate each selected paper using the NICE instruments. Disagreements between reviewers will be resolved by discussion. A third reviewer will be involved, if necessary.

Strategy for data synthesis

A meta-analysis is not appropriate as a synthesis strategy given the small number of studies and the large diversity of research designs. Instead, we will conduct a narrative synthesis to identify the primary barriers and enablers of effective maintenance of WASH infrastructure in schools. We will also discuss the types of interventions that have been implemented in this field, and emphasize the conditions under which they were successful or unsuccessful. PRISMA guidelines will be followed when reporting findings from this review.

Analysis of subgroups or subsets

No planned analysis of subgroups due to the limited number of selected papers.

Contact details for further information

Christine Pu
cjpu@stanford.edu

Organisational affiliation of the review

Stanford University

Review team members and their organisational affiliations

Ms Christine Pu. Stanford University
Dr Jenna Davis. Stanford University
Dr Gary Darmstadt. Stanford University

Type and method of review

Intervention, Narrative synthesis, Synthesis of qualitative studies, Systematic review

Anticipated or actual start date

01 April 2020

Anticipated completion date

01 October 2020

Funding sources/sponsors

World Vision

Conflicts of interest

Language

English

Country

United States of America

Stage of review

Review Ongoing

Subject index terms status

Subject indexing assigned by CRD

Subject index terms

MeSH headings have not been applied to this record

Date of registration in PROSPERO

15 August 2020

Date of first submission

15 July 2020

Stage of review at time of this submission [1 change]

Stage	Started	Completed
Preliminary searches	Yes	Yes
Piloting of the study selection process	Yes	Yes
Formal screening of search results against eligibility criteria	Yes	Yes
Data extraction	Yes	Yes
Risk of bias (quality) assessment	Yes	Yes
Data analysis	Yes	Yes

The record owner confirms that the information they have supplied for this submission is accurate and complete and they understand that deliberate provision of inaccurate information or omission of data may be construed as scientific misconduct.

The record owner confirms that they will update the status of the review when it is completed and will add publication details in due course.

Versions

15 August 2020
15 November 2020
